# The impact of pharmacotherapy on sexual function in female patients being treated for idiopathic overactive bladder: a systematic review

**DOI:** 10.1186/s12905-024-03103-1

**Published:** 2024-05-16

**Authors:** Christopher Neal Bruce Evans, Anja Badenhorst, Frans Jacob Van Wijk

**Affiliations:** 1 Surgery Masters in Urology, the University of Edinburgh (Edinburgh Surgery Online, Deanery of Clinical Sciences) and Life Groenkloof Hospital, Suite 209, Life Groenkloof Hospital, 50 George Storrar Drive, Groenkloof, Pretoria, 0181 South Africa; 2 General Practitioner, University of Pretoria, Pretoria, South Africa; 3 Private Uro-Gynaecologist/Urologist, Pelvic Wellness Unit, The Urology Hospital, Pretoria, South Africa

**Keywords:** Overactive bladder, Female, Sexual function

## Abstract

**Background:**

Overactive bladder (OAB) is a condition defined by urgency with or without incontinence which disproportionately affects female patients and has a negative impact on sexual enjoyment and avoidance behaviour. Pharmacotherapy can be considered one of the main options for treating OAB. This research set out to determine the impact of pharmacotherapy on sexual function in females with OAB.

**Methods:**

This research used the robust methodology of a systematic review. The clinical question was formulated using the PICO (population, intervention, control, and outcomes) format to include females being treated with pharmacotherapy (anticholinergics or beta-3 adrenergic agonists) for idiopathic OAB with the use of a validated questionnaire assessing self-reported sexual function at baseline and post-treatment. The review incorporated the MEDLINE, PubMed and EMBASE databases. The AMSTAR 2 (A Measurement Tool to Assess Systematic Reviews) appraisal tool was used to guide the review process. Two reviewers worked independently in screening abstracts, deciding on the inclusion of full-texts, data extraction and risk of bias assessment.

**Results:**

In female patients with OAB, pharmacotherapy does seem to offer at least partial improvement in self-reported sexual function outcomes after 12 weeks of therapy. Still, the value of this finding is limited by an overall poor quality of evidence. Patients with a higher degree of bother at baseline stand to benefit the most from treatment when an improvement within this health-related quality of life domain is sought.

**Conclusion:**

This research should form the basis for a well-conducted randomized controlled study to accurately assess sexual function improvements in females being treated with pharmacotherapy for OAB.

**Supplementary Information:**

The online version contains supplementary material available at 10.1186/s12905-024-03103-1.

## Background

Overactive bladder is a common condition which impacts quality of life within the spheres of physical, social, psychological, and sexual health [1, 2]. The EpiLUTS highlighted the association of OAB symptoms, and its negative impact on sexual enjoyment and avoidance behaviour, with both decreased arousal and desire being prevalent amongst respondents with these symptoms [2, 3]. To further highlight this association *Proietti S, *et al*.*, showed decreased sexual enjoyment in patients with wet and dry OAB of 25% and 20%, respectively, versus only 2% of patients with no bladder symptoms [3]. In a prospective case–control study by *Naumann G, *et al*.*, OAB had a greater adverse impact on sexual health than stress incontinence [4].

There have been several well-powered and well-designed double-blinded randomized controlled trials (RCTs) published in which primary endpoints of efficacy – namely urinary episodes/24 h, urgency incontinence episodes/24 h and mean voided volume – were assessed [5–7]. Although there is no dispute that these are the primary endpoints which are required to be assessed, due to the lack of a standardized tool in initial assessment and reporting on improvement, very few of these studies give us an indication of the impact OAB has on sexual function and outcomes following treatment.

Anticholinergics are widely used for patients with idiopathic overactive bladder [8, 9]. These medications work by competitively blocking the muscarinic receptors within the detrusor muscle of the bladder [8]. The most ubiquitous muscarinic receptors within the detrusor are the M2 and M3 receptors [10]. Commonly reported side effects of the class of medication include somnolence, cognitive decline, blurred vision, dry mouth, and constipation [9]. Interestingly, although both M2 and M3 receptors have been found via real-time PCR analysis of human vaginal muscularis tissue taken at the time of hysterectomy, the effect on female sexual function of these medications remains poorly reported [11].

The use of beta-3 adrenergic receptor agonists followed from in vitro studies where mRNA expression of β1, β2 and beta-3 adrenergic receptors within the human detrusor was shown [12]. Beta-3 adrenergic receptors are presumed to be the main mediator of detrusor relaxation and have a concentration-dependent effect [12, 13]. *Coelho A, *et al*.,* postulated that relaxation of the detrusor may be due to an inhibition of acetylcholine release, thereby dampening detrusor contractions mediated by the parasympathetic nervous pathway [14].

Within the primary domains of reported OAB outcomes (voids per day and leakage), anticholinergic medication has been shown in a meta-analysis to provide a benefit as compared to placebo [15]. Likewise concerning beta-3 agonists, a meta-analysis comparing mirabegron to placebo showed that there was a statistically significant improvement or cure of urgency urinary incontinence, fewer voids per day or number of urgency episodes and an increase in the voided volume in participants of RCTs who were taking mirabegron [16]. In terms of comparing the clinical efficacy of beta-3 agonists to anticholinergics, there is no clear benefit of either group of agents when used as monotherapy, with the most notable difference being that of the side effect profile [17, 18].

Sexual activity is not limited to vaginal penetration or intercourse but can include any act causing sexual arousal, whether it be solitary or between people. Sexual function in females is an important predictor of general well-being and satisfaction within a relationship, regardless of age [19]. Female sexual function can be classified into the domains of desire, arousal, lubrication, orgasm, and satisfaction. These have been incorporated into the Female Sexual Function Index (FSFI), which included the domain of pain, into the formulation of a validated questionnaire [20].

The impact of OAB on sexual function can be approached considering two paradigms of causation. OAB may cause a direct impact on sexual behaviour (incontinence associated with sexual intercourse, pain during intercourse or interruption of sex due to urgency) or it could be due to consequences related to psychological manifestations of having OAB which may lead to a negative self-image, sexual satisfaction (embarrassment, fear of leaking and fear of worsening symptoms post sexual activity) which ultimately would lead to avoidance behaviour [21]. If OAB symptoms were controlled via pharmacotherapy means, improvement in sexual satisfaction would presumably follow.

A second important consideration, however, would require an understanding of physiology and receptors within both the vagina and bladder and how pharmacotherapy may impact sexual function when OAB is being treated. Central nervous system and/or peripheral sensory stimulation can induce genital arousal, both having a common effect in modulating and activating the autonomic nervous system with initially sacral parasympathetic motor neurons inducing genital vaso-congestion and lubrication followed later by diffuse sympathetic nervous system discharge [22]. This explains the typical increase in blood pressure and heart rate observed before orgasm [22]. Due to the close anatomical relationship between the bladder and the vagina, and the commonality in terms of neuro-receptors, medications which modulate the autonomic nervous system response intended for treating OAB, may have an impact on sexual function [11, 22].

The primary aim of this review was thus to assess whether there was sufficient evidence to determine whether first-line pharmaceutical medications improve female sexual function in patients with overactive bladder syndrome. A secondary aim was to assess whether there are differing, medication-specific improvements within certain domains of sexual function, which ensure tailoring in medication selection in patients where specific domains are maximally affected.

## Methods

Although a formal online, published protocol is not available, as this systematic review was done in support of the primary author’s Master of Surgery (Urology), through the University of Edinburgh, a protocol outlining the design and methodology was submitted as an initial project phase. This review was thus done according to a pre-specified search strategy and data synthesis plan.

The PICO (Population, Intervention, Comparison and Outcome) approach was used to formulate an appropriate question as described below in Table 1.

**Table 1:** The pico (Population, Intervention, Comparison, and outcome) framework used to formulate the search strategy

Population (3)	Intervention (20)	Comparison (2)	Outcome (5):
Adult (> 18 years) Female Idiopathic overactive bladder	Pharmacotherapy: Antimuscarinics Beta-3 adrenergic agonist	Baseline Placebo	Female Sexual Function Index Sexual Quality of Life–Female questionnaire The Arizona Sexual Experience Scale “Personal Relationships Domain” from Kings Health Questionnaire International Consultation on Incontinence Modular Questionnaire – Female Sexual Matters associated with LUTS

### Study selection

Inclusion of both RCTs and non-randomized studies of therapeutic interventions (NSTIs) was required. RCTs published had mixed gender cohorts and most often incorporated the King’s Health Questionnaire (KHQ). At the time of review the FSFI, which is a better female sexual function assessment tool, had only been used in NSTIs.

### Inclusion criteria

Female patients; idiopathic overactive bladder, health-related quality of life with sexual function in the questionnaire: Female Sexual Function Index (FSFI), Personal relationships domain in the King's Health Questionnaire (KHQ), the Arizona Sexual Experience Scale (ASEX), Sexual Quality of Life–Female questionnaire (SQOL-F) and the International Consultation on Incontinence Modular Questionnaire – Female Sexual Matters associated with Lower Urinary Tract Symptoms (ICIQ-FLUTSsex); pharmacotherapy with beta-3 adrenergic receptor agonist, anticholinergic/muscarinic antagonist; and studies published in English language.

### Exclusion criteria

Male; Onabotulinum A; intravesical therapy; posterior tibial nerve stimulation; neurogenic bladder; children (< 18 years); alternative or herbal therapies; yoga; pelvic muscle floor training; surgery; anonymous author; biofeedback/bladder training; dementia; sacral neuromodulation; and animal studies.

### Information and search strategy

This study was done using the AMSTAR 2 appraisal tool as a guiding framework in conducting this systematic review [23]. The databases used to search for articles included in this study were Ovid MEDLINE, PubMed and EMBASE. The search strategy focused on including all relevant articles which used pharmacotherapy in the treatment of idiopathic overactive bladder. For these, the predefined medical subject headings of “Urinary Bladder, Overactive”, “Cholinergic Antagonists”, “Adrenergic Beta-Agonists”, and “Muscarinic Antagonists” were used. Included is the Ovid MEDLINE search strategy:

Ovid MEDLINE ® ALL < 1946 to March 31, 2022 >

**Table:** 

1	Cholinergic Antagonists/	5581
2	Adrenergic Beta-Agonists/	17764
3	Muscarinic Antagonists/	9347
4	1 or 2 or 3	31895
5	Urinary Bladder, Overactive/	5507
6	4 and 5	1327

### Article selection

Once articles were retrieved following the database search, these were imported to the Covidence website, which was used as a screening and study selection platform [24]. This platform allowed the two reviewers to independently assess abstracts for trial design and interventions in an idiopathic overactive bladder cohort, where a sexual function questionnaire was incorporated. Strict adherence to the inclusion and exclusion criteria was followed while assessing abstracts. Where there were differences in the decision to include or exclude, both reviewers met in person and discussed the differing opinions with agreement being reached before progressing to the full text review. References of included articles were also assessed and where appropriate, original articles were included in the Covidence database for screening.

### Data collection

Data was collected by both reviewers working independently and populating specifically designed Excel spreadsheets for each specific sexual function questionnaire. Data collected included trial type, the number enrolled in the study and completed, age, percentage of female participants, drug used including dosage, baseline sexual function symptom score and symptom score at completion, and the trial duration.

### Risk of bias assessment

Risk of bias (RoB) was assessed according to the trial designs, namely RCTs and NSTIs. For the NSTIs, the ROBIN-I (Risk of Bias in Non-randomized Studies) risk assessment tool was used as an evaluation tool, and for the RCTs the RoB2 [25, 26]. Based on the certainty of evidence a Grading of Recommendations Assessment, Development and Evaluation (GRADE) approach was followed to summarize the findings and provide a recommendation on the outcome and guide clinical decision-making where feasible [27].

### Assessment of heterogeneity

Trials were assessed after data collection and visually inspected to assess whether there were fundamental concerns with differences in the trials evaluated, which would compromise the statistical assimilation of data. Once this was done, data was considered using the I^2^ statistic as described by *Higgins JP, *et al*.* [28].

## Results

### Determination of studies to include

Reasons for full-text exclusion: 10 Patient data included in another study (post hoc), 6 Duplicate, 3 Wrong intervention, 2 Wrong outcomes, 2 Wrong study design, 2 Abstract (no full text), 2 Not English language, 2 Observational/post-marketing surveillance, 1 Intervention ambiguous, critical bias.

**Fig. 1:**
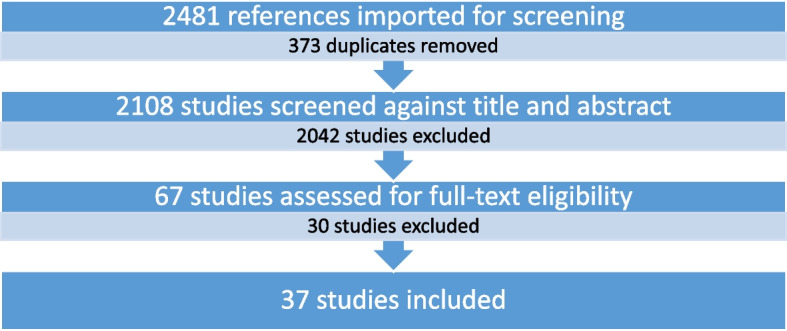
Prisma (Preferred Reporting Items for Systematic Reviews and Meta-Analyses) study flow diagram

**Fig. 2:**
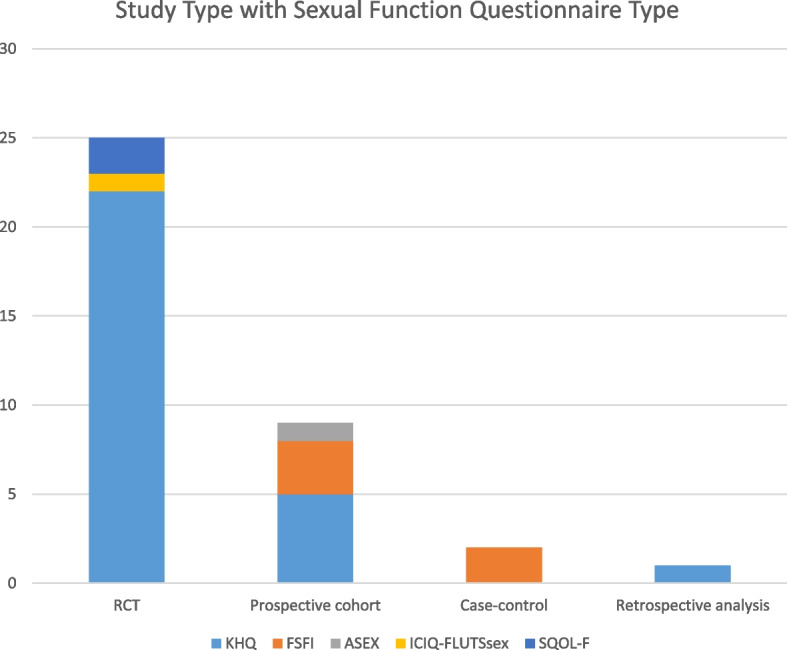
Break down of included studies

Thirty-seven studies were included after full texts were assessed for appropriateness after 30 were excluded with reasons summarized in Fig. 1. Of the included studies, 25 were randomized controlled trials, of which 22 used the KHQ, 2 used the SQOL-F and one trial used the ICIQ-FLUTS. In terms of the non-randomized studies of therapeutic interventions 6 used the KHQ, 5 used the FSFI, and 1 used the ASEX. A graphical representation of the study type with sexual function questionnaire type is shown in Fig. 2.

Pooled studies were only included where the data was scrutinized and found to not have been incorporated as a replication of another study reviewed and where the original article had not published the desired data.

### Risk of bias

The risk of bias was assessed according to the selected articles’ main aims and how this data was presented. It is worth noting however, that the secondary domains of KHQ data concerning personal relationships were underreported in a large portion of the participants and as such would introduce a significant element of bias with regards to using this data on which to draw conclusions. Due to the bias that may be introduced in assessing and incorporating secondary outcomes (in sexual function), the reviewers modified the RoB assessments to include a section specifically on the completeness and quality of this data.

Presented are Tables 2 and 3 which highlight both the summary of the risk of bias for randomized controlled trials and non-randomized studies of therapeutic intervention using the RoB2 and ROBIN-I risk assessment tools respectively [25, 26]. This was done by both reviewers. Included in Appendix A is the complete risk of bias assessments.

**Table 2:**
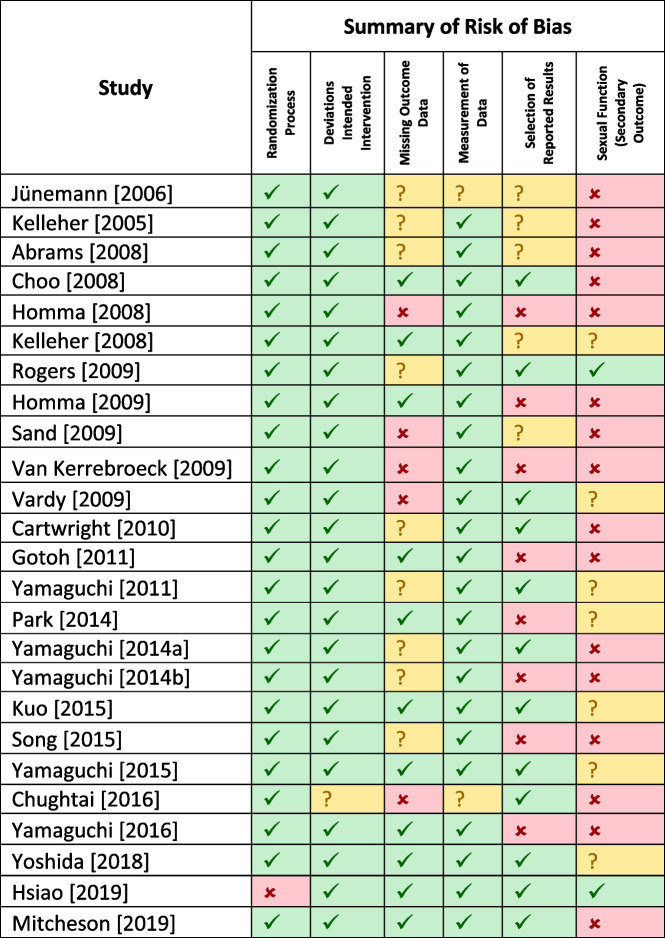
RoB2 for RCTs

Table 2 is a tabulated summary of the risk of bias using the RobB2 assessment tool [26]. The following key provides the conclusion/ summary on the assessment for risk of bias: Low =  ; some concern =  ; high =  [29–52]

**Table 3:**
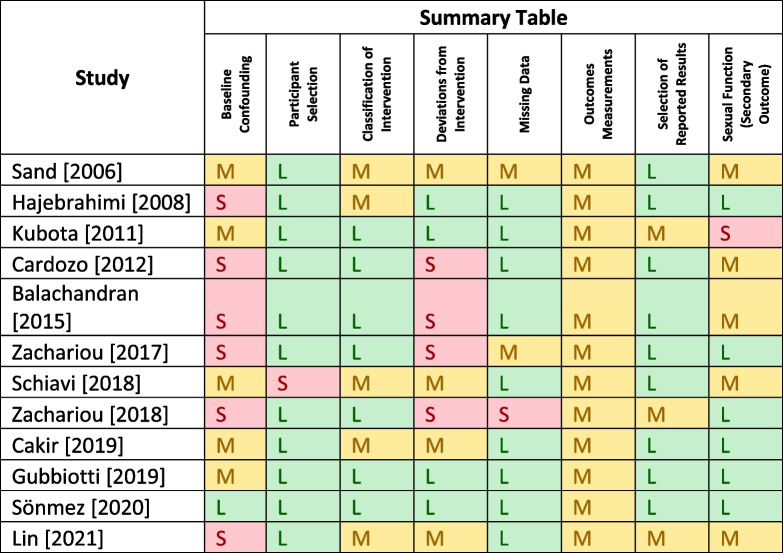
Robin-I for NSTI

Table 3 is a tabulated summary for the risk of bias using the ROBIN-I assessment tool [25]. The following key provides the conclusion of the risk of bias assessment: low =  ; moderate =  ; serious =  [53–61]

### RoB2 for RCTs

#### ROBIN-I for NSTI

##### Declared potential conflict of interest

Studies in which it was declared that there was an affiliation or funding was received from a pharmaceutical company are referenced: [29–40, 42–46, 48, 50–52, 56, 57, 62, 63].

### Sexual function analysis tools

#### King’s Health Questionnaire (KHQ)

This health-related quality of life (HRQOL) assessment tool was first developed by *Kelleher CJ, *et al*.* in 1997 as a rapid, reliable and validated questionnaire after realising the need to assess the impact and change of HRQOL in a trial setting [64]. The KHQ has undergone several revisions and adaptations for different languages and cultures, with the basic construction being that of a 27-item questionnaire that covers 10 domains [41, 42, 45, 53, 62]. The aspect of the KHQ that addresses sexual function outcomes is the personal relationships domain which specifically enquires about the degree of bother the bladder condition has on the relationship with the patient’s partner, the degree of impact on sex life and the impact on family life [34]. The personal relationships domain is assessed using a degree of severity score (not at all, a little, moderately, or a lot) to describe the degree to which the lower urinary tract dysfunction impacts this domain [62]. Each domain is scored out of 100 with 0 being no impairment and 100 being the greatest impairment with respect to the domain [62]. For most domains, a change of greater than or equal to 5 is classified as the minimally important difference, which suggests a clinically meaningful improvement [62].

The King’s Health Questionnaire was the most widely used within well-designed (for primary outcomes) randomized controlled trials, yet as a secondary outcome there were inconsistencies with reported data. Included is a summary of the studies (Table 4) which used the KHQ [29–32, 34–37, 39–46, 48–51, 55–58, 61–63, 65], which met the inclusion criteria for this systematic review.

**Table 4:** Studies using KHQ data summary

Study	Trial design	Control	Duration	Drug and Dose	Female Participants	Percentage Female Participants	Age		% PR response	Baseline PR Score	PR improvement	Method	Personal Relationship Outcome	Statistical significance	Comparator
							**Mean**	**SD**		**Mean**					
Kelleher [2005] [30]	RCT	Yes	12 weeks	Placebo					63.9%		-9.7	Mean change from baseline	Improved		Baseline
*pooled				Solifenacin 5 mg					67.5%		-8.7	Mean change from baseline	Equivocal	Not statistically significant	Placebo
				Solifenacin 10 mg					64.1%		-9.3	Mean change from baseline	Equivocal	Not statistically significant	Placebo
Jünemann [2006] [29]	RCT	Yes	32 days	Placebo	183	90.6%							Not reported		
				Propiverine IR 15 mg b.d	353	89.4%							Not reported		
				Propiverine ER 30 mg	348	89.0%							Not reported		
Sand [2007] [62]	Prospective cohort	No	Up to 6 months	Oxybutinin TDS 3.9 mg/d	2508	87.2%	62.5	14.8	68.9%	20.6	-6	Adjusted mean change	Improved	Statistically significant	Baseline
Abrams [2008] [31]	RCT	Yes	12 weeks	Placebo	331	85.3%	56			26.5					
				Darifenacin 7.5 mg	288	85.5%	57			24.1			Improved	Not statistically significant	Baseline
				Darifenacin 15 mg	281	84.1%	57			26.8			Improved	Not statistically significant	Baseline
Choo [2008] [32]	RCT	Yes	12 weeks	Solifenacin 5 mg	90	84.1%	53.07	10.5	65.4%		-9.31	Adjusted mean change	Improved	Not statistically significant	Tolterodine
				Solifenacin 10 mg	83	74.8%	52.65	12.7	61.3%		-7.08	Adjusted mean change	Worsened	Not statistically significant	Tolterodine
				Tolterodine 2 mg b.d	88	79.3%	53.05	12.2	60.9%		-7.78	Adjusted mean change	Improved	Not calculated	Baseline
Homma [2008] [33]	RCT	Yes	12 weeks	Placebo	69	72.6%	61.9	11.8					Not reported		
				Imidafenacin 0.1 mg	68	74.7%	62.5	13.0					Not reported		
				Imidafenacin 0.2 mg	63	67.7%	64.5	13.5					Not reported		
				Imidafenacin 0.5 mg	50	65.8%	63.6	12.9					Not reported		
Kelleher [2008] [34]	RCT	Yes	12 weeks	Placebo	430	77.6%	57		100.0%		-5.9	Mean change from baseline	Improved	Not statistically significant	Baseline
*Pooled data				Tolterodine ER 4 mg	227	78.3%	58		100.0%		-10	Mean change from baseline	Improved	Not statistically significant	Baseline
				Fesoterodine 4 mg	434	78.3%	58		100.0%		-7.8	Mean change from baseline	Improved	Not statistically significant	Baseline
				Fesoterodine 8 mg	452	79.9%	57		100.0%		-9.6	Mean change from baseline	Improved	Statistically significant	Placebo
Homma [2009] [35]	RCT	Yes	12 weeks	Placebo	125	87.4%	58	13.5							
				Imidafenacin 0.1 mg b.d	278	87.4%	57.7	12.7					Improved	Statistically significant	Placebo
				Propiverine 20 mg	257	84.3%	59.8	11.9					Not reported		
Sand [2009] [36]	RCT	Yes	12 weeks	Placebo	505	100.0%	58.2	0.5							
*subgroup analysis females				Trospium ER 60 mg	484	100.0%	59.2	0.6					Not reported	Not statistically significant	Placebo
Van Kerrebroeck [2009] [37]	RCT	Yes	12 weeks	Placebo	401	82.3%	61	14.0	65.3%		-3.5	Mean change from baseline			
				Tolterodine ER 4 mg	412	82.4%	60	14.0	61.8%		-5.8	Mean change from baseline	Improved	Not statistically significant	Placebo
Cartwright [2011] [51]	RCT	Yes	4 weeks	Placebo	48	100.0%	50.5	13.7	100.0%					Not statistically significant	
				Oxybutinin TDS 3.9 mg/d	48	100.0%	53.1	14.5	100.0%				Improved	Not statistically significant	Baseline
Gotoh [2011] [39]	RCT	Yes	12 weeks	Placebo	207	76.7%	58.7	14.1							
				Propiverine 20 mg	216	76.1%	56.6	13.6					Improved	Not statistically significant	Placebo
Kubota [2011] [55]	Prospective cohort	No	12 weeks	Propiverine 10 mg b.d	58	61.1%	68.6	14.8					Improved	Not statistically significant	Baseline
Yamaguchi [2011] [40]	RCT	Yes	12 weeks	Placebo	251	78.9%	56.7	13.5	76.5%	25.34	-8.33	Mean change from baseline			
				Fesoterodine 4 mg	251	78.4%	57.2	14.2	77.0%	27.73	-16.11	Mean change from baseline	Improved	Not calculated	Placebo
				Fesoterodine 8 mg	255	81.5%	58.8	13.4	74.6%	24.2	-9.98	Mean change from baseline	Improved	Not calculated	Placebo
Cardozo [2012] [56]	Prospective cohort	No	12 weeks	Fesoterodine 4 mg	263	79.5%	60.3	12.4	100.0%		-15.2	Mean change from baseline	Improved	Not calculated	Baseline
Park [2014] [41]	RCT	Yes	12 weeks	Imidafenacin 0.1 mg b.d	57	85.1%	58.31	11.5	100.0%				Improved	Statistically significant	Baseline
				Propiverine 20 mg	55	85.9%	56.13	11.3	100.0%				Improved	Statistically significant	Baseline
Yamaguchi [2014a] [42]	RCT	Yes	12 weeks	Placebo	344	92.2%	56.2	13.2	52.0%						
				Oxybutinin patch 35cm^2^	502	90.5%	55.4	12.4	56.0%				Improved	Statistically significant	Placebo
				Propiverine 20 mg	478	85.5%	55.6	12.5	56.2%				Improved	Not statistically significant	Placebo
Yamaguchi [2014b] [43]	RCT	Yes	12 weeks	Placebo	310	84.2%	58.2	14.2	76.1%	9.3					
				Mirabegron 50 mg	311	84.3%	58.3	13.9	76.0%	9.9			Improved	Not statistically significant	Placebo
				Tolterodine ER 4 mg	304	82.6%	58.3	13.7	79.5%	7.9			Improved	Not calculated	Placebo
Balachandran [2015] [57]	Prospective cohort	No	6 weeks	Mirabegron 50 mg	67	100.0%	59.3	12.2		33.16			Improved in responders	Statistically significant	Baseline
Kuo [2015] [44]	RCT	Yes	12 weeks	Placebo	225	69.7%	55.3	13.6	80.5%	23.25	-4.3	Mean change from baseline	Improved	Not statistically significant	Baseline
				Mirabegron 50 mg	228	67.5%	54.3	14.2	80.5%	24.01	-3.96	Mean change from baseline	Improved	Not statistically significant	Baseline
				Tolterodine ER 4 mg	213	64.0%	53.9	14.5	80.7%	25.9	-5.8	Mean change from baseline	Improved	Not statistically significant	Baseline
Song [2015] [45]	RCT	Yes	12 weeks	Placebo	51	70.8%	58.35	12.4					Equivocal	Not statistically significant	Baseline
				Tarafenacin 0.2 mg	48	62.3%	59	10.6					Worsened	Not statistically significant	Baseline
				Tarafenacin 0.4 mg	50	65.8%	60.18	10.8					Improved	Not statistically significant	Baseline
Yamaguchi [2015] [46]	RCT	Yes	12 weeks	Placebo	169	80.1%	55.7	12.9	79.6%	8.8	-0.8	Mean change from baseline			
				Mirabegron 25 mg	168	80.4%	54.9	13.6	79.9%	10	-3.5	Mean change from baseline	Improved	Not calculated	Placebo
				Mirabegron 50 mg	177	85.1%	56.2	13.6	81.5%	10.8	-2.6	Mean change from baseline	Improved	Not calculated	Placebo
				Mirabegron 100 mg	172	83.1%	56.9	13.3	80.0%	10	-3.2	Mean change from baseline	Improved	Not statistically significant	Placebo
Yamaguchi [2016] [63]	RCT	Yes	8 weeks	Placebo	130	88.4%	56.2	13.7	76.9%						
				Oxybutinin patch 73.5 mg	118	88.7%	53	14.0	78.9%				Improved	Statistically significant	Placebo
				Oxybutinin patch 105 mg	115	82.1%	55.3	14.7	71.2%				Improved	Not statistically significant	Placebo
Schiavi [2018] [58]	Retrospective analysis	Yes	12 weeks	Solifenacin 5 mg	168	100.0%	58.34	6.1	100.0%	48.16			Improved	Statistically significant	Baseline
				Mirabegron 50 mg	174	100.0%	59.12	5.2	100.0%	47.82			Improved	Statistically significant	Baseline
Yoshida [2018] [48]	RCT	Yes	12 weeks	Placebo	333	90.2%	58.9	11.8	82.7%	9.39	-2.57	Adjusted mean change			
				Vibegron 50 mg	334	90.3%	58	11.8	82.4%	8.02	-4.65	Adjusted mean change	Improved	Statistically significant	Baseline
				Vibegron 100 mg	330	89.7%	58.7	11.1	84.0%	10.49	-3.88	Adjusted mean change	Improved	Not statistically significant	Baseline
				Imidafenacin 0.1 mg bd	105	89.7%	59.7	12.4	80.3%	11.36	-4.01	Adjusted mean change	Improved	Not calculated	Baseline
Hsiao [2019] [49]	RCT	6 months Rx	3 months	Solifenacin 5 mg	91	100.0%	59.2	13.7	100.0%	29.5	-11.1	Mean change from baseline	Improved	Not calculated	Baseline
			6 months	Solifenacin 5 mg	91	100.0%	60	12.8	100.0%	26.1	-6.7	Mean change from baseline	Improved	Not calculated	Baseline
Mitcheson [2019] [50]	RCT	Yes	8 weeks	Placebo	185	90.2%	57.8	9.5							
				Vibegron 50 mg	129	86.0%	60.3	8.7			-6.72	Adjusted mean change	Improved	Not statistically significant	Placebo
				Vibegron 100 mg	236	90.4%	59	9.2			-4.35	Adjusted mean change	Improved	Not statistically significant	Placebo
Sönmez [2020] [61]	Prospective cohort	Yes	12 weeks	Sodium bicarbonate 4 g b.d	31	100.0%	55.6	15.9	100.0%	36.3	-20.5	Calculated mean change	Improved	Statistically significant	Baseline
				Solifenacin 5 mg	28	100.0%	48.3	14.6	100.0%	32.9	-14.15	Calculated mean change	Improved	Statistically significant	Baseline

*indicates where secondary publication using pooled or subgroup analysis was performed with no duplication of data ensured

Analysis of the data from the KHQ results showed that there is great heterogeneity within the percentage of female patients included in studies with an average of 84.5% across all studies analysed with a range of 61.1% to 100%. The only study which included a specific subgroup analysis was that of *Cordozo L, *et al*.* where the personal relationships domain of the KHQ improvement on pharmacotherapy was greater in female patients (-16.7, SD 28.1) than in male patients (-10.6, SD 20.6) treated with fesoterodine [56]. Further of note, the percentage of participants who completed the personal relationships domain of the KHQ in relation to the general health perception was 76.7% of the total population reviewed. As there was missing data and a lack of accurate reporting allowing for female-specific subgroup analysis, corresponding authors were emailed requesting the required data (a communication log sheet is provided as Appendix B). Unfortunately, no additional information could be accessed to improve the quality of data and hence analysis thereof.

Some data sets did not include baseline values which impacts the interpretation of the results, as a cohort with a poor baseline (i.e. higher KHQ score) would stand to gain the most in terms of benefit [30, 34, 37, 50]. It is also important to note that in some studies KHQ data was not reported on at all, with only a comment on benefit and whether this reached statistical significance within certain domains [29, 35, 36, 45, 65]. The heterogeneity within gender breakdown, the range of those who completed the KHQ personal relationships domain (range 51.2 to 100%) in addition to the diverse drugs used with statistically differing endpoints (response to placebo vs. response from baseline; response reported as a minimum important difference, KHQ improvement in relation to OAB symptoms improvement, adjusted mean change vs. mean change), the diverse manner in which results were reported and the poor completion of the personal domain section of the KHQ (the only domain that specifically addresses sexual outcomes) mean that the conditions to proceed with a meta-analysis are not met.

#### Female Sexual Function Index (FSFI)

The FSFI, developed by *Rosen R, *et al*.,* has become the standard questionnaire as quoted by *Sand M, *et al*.* in assessing female sexual function [20, 66]. The FSFI is a 19-item questionnaire that is used to categorize female sexual function over the past four weeks into the six domains of desire, arousal, lubrication, orgasm, satisfaction, and pain [20]. Each question is scored on a 5-point Likert scale, with a factor used to weight domains with total scores being calculated ranging from 2.0 to 36.0 with lower scores reflecting a worse sexual function [67, 68]. A clinical cut-off of 26.55 has been established as the threshold to classify a patient as having sexual dysfunction [68].

**Fig. 3:**
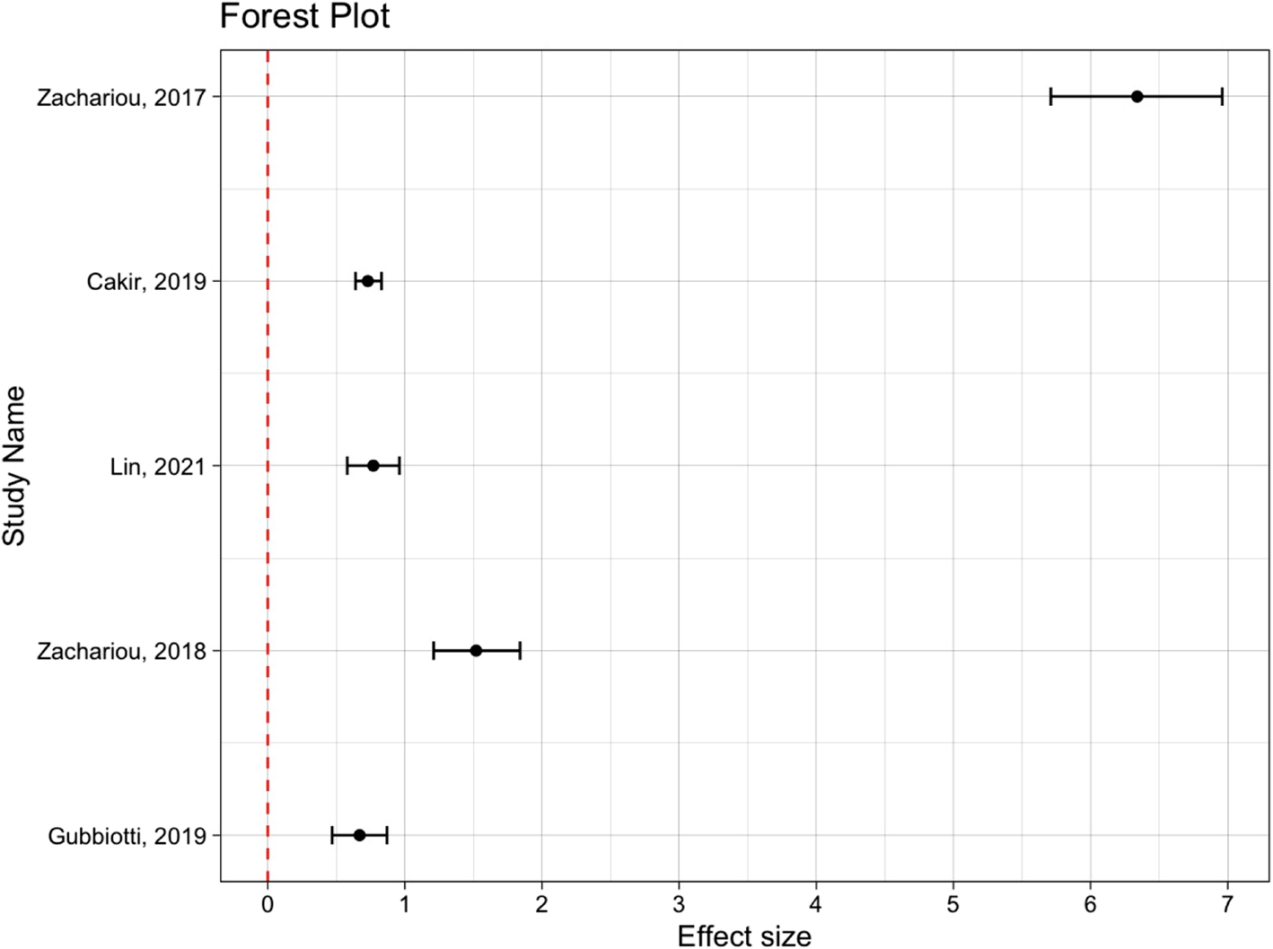
Forest plot of impact of interventions

The studies included that used the FSFI were non-randomized studies of therapeutic intervention [59, 60, 69–71]. Although no standard reporting system is agreed upon for non-randomized trials, a Forest plot was generated for appropriate graphic interpretation using the Meta-Essentials tool [72]. The workbook using differences between dependent groups with continuous data was utilised. Means, standard deviations and number treated were used with the correlation coefficient (r), which was not reported, being taken from the work of *Rosen R, *et al. (*r* = 0.8) [20, 72]. From the calculations, I^2^ was shown to be 98.8% which would signify significant heterogeneity [28]. As non-randomized studies of therapeutic intervention lack a gold standard for reporting and synthesizing data, and due to the high level of heterogeneity as calculated using the I^2^ statistic, a Forest plot (Fig. 3) is provided for summary of the analysis, although no pooled effect is shown due to the limitations as stated above [73].

For the analysis of specific domain improvements, Appendix C can be viewed.

Although direct comparison between these studies is not feasible due to differences in study designs and medications, it is interesting to note that across all trials desire, arousal, orgasm, satisfaction and overall FSFI score improvement reached statistical significance (Table 5) [59, 60, 69–71]. In the published outcomes of *Gubbiotti M, *et al*.,* mirabegron was not shown to result in a statistically significant improvement within the pain domain, and in the study by *Cakir SS, *et al*.,* no statistically significant improvement within the lubrication domain was shown [60, 69]. These results show improvement from baseline, with patients not blinded or randomized, which results in the weighting of evidence for these findings being weak with inherent baseline confounding.

**Table 5:** FSFI Summary comparison – baseline to post treatment

Study	Design	Control	Drug	Dose	N treated	Baseline		Post treatment		
						**FSFI**	**SD**	**FSFI**	**SD**	***P*-value**
Zachariou [2017] [71]	Prospective cohort	OAB	Tolterodine	ER 4 mg	85	17.4	1.2	26.5	1.5	< 0.01
Cakir [2019] [60]	Case–control	Healthy	Anticholinergics	Missing data	216	21.47	3.22	23.72	2.61	< 0.01
Lin [2021] [70]	Case–control	Healthy	Tolterodine	2 mg b.d	55	13.25	7.03	18.84	3.98	< 0.001
Zachariou [2018] [59]	Prospective cohort	OAB	Mirabegron	50 mg	35	20.3	3.8	26.6	4.2	< 0.001
Gubbiotti [2019] [69]	Prospective cohort	None	Mirabegron	50 mg	50	18.9	4.3	21.9	4.5	< 0.0001

#### Sexual Quality of Life – Female (SQOL-F)

The SQOL-F (ESM) is a quality of life assessment tool developed to assess female sexual function, where answers are scored on a 6-point scale from strongly agree to strongly disagree with a higher score reflecting a better quality of life [74]. This assessment tool has been internally and externally validated and primarily focuses on quality of life as experienced in terms of social, emotional, psychological, and physical consequences [74, 75]. Studies assessing response to pharmacotherapy within a population of OAB patients using the SQOL-F questionnaire were conducted by *Rogers R, *et al*.,* and *Chugtai B, *et al*.* (Table 6) [47, 52].

**Table 6:** Summary of studies using SQOL-F

Study	Trial design	Duration	Drug and dose	N Enrolled	N Females	Age	Baseline SQOL-F		Trial End SQOL-F		Outcome	Statistical significance	Comparator
						**Mean**	**Mean**	**SD**	**Mean**	***P***-value			
Rogers [2008] [52]	RCT	12 wks	Placebo	211	211	47	69.2	23					
			Tolterodine 4 mg	202	202	49	69.6	23.1		< 0.01	Improved	Statistically significant	Placebo
Chugtai [2016] [47]	RCT	12 wks	Fesoterodine	12	12	55.4	51		81	0.02			
			Fesoterodine + topical oestrogen	11	11		56		99	0.0003	Improved	Not statistically significant	Festerodine

The study by *Chugtai B, *et al*.,* is underpowered to provide any statistically significant findings and uses the addition of topical oestrogens which was one the only study assessed during this review that used this management [47]. Participants and observers were unblinded as to which arms the study participants were in [47]. Data of the SQOL-F in the *Rogers R, *et al*.* paper used graphical representation illustrating improvement and confirming the statistical significance of this finding [52].

#### Arizona Sexual Experience Scale (ASEX)

Within the studies in this systematic review, the only study which used the ASEX to assess sexual function outcomes in patients undergoing pharmacotherapy for the management of OAB was by *Hajebrahimi S, *et al*.* [54]. The ASEX is a multi-domain symptom score which includes desire, arousal, vaginal lubrication, orgasm and orgasm satisfaction [54]. The score ranges from 0 to 30 with higher values indicative of greater sexual dysfunction [76]. In the study reviewed, 30 female patients received treatment with tolterodine IR 2mg b.d. for OAB [54]. The ASEX total score at baseline was 18.79 (mean) ± 4.92 (SD), which improved to 11.52 ± 4.96 after 3 months of treatment which was shown to be statistically significant (*p* < 0.01) [54].

#### International Consultation on Incontinence Modular Questionnaire – Female Sexual Matters associated with Lower Urinary Tract Symptoms (ICIQ-FLUTSsex)

This is a validated questionnaire available through the International Consultation on Incontinence that has been externally validated [77]. It has four questions, the first two scored from 0 to 3 and the last from 0 to 4, with a higher score indicating a higher degree of sexual dysfunction [38, 77]. The questions include pain or discomfort due to vaginal dryness, the extent to which sex life has been spoilt, pain associated with sexual intercourse and urine leakage during sexual intercourse with each question having an additional degree of bother score [38, 77].

In the VIBRANT study, a multicentre randomized, double-blinded trial assessing the efficacy of solifenacin (5mg with dose escalation at 4 weeks) daily episodes of urgency, incontinence and frequency were significantly improved as compared to placebo [38]. The ICIQ-FLUTSsex overall symptom score improvement did favour treatment with solifenacin over placebo but did not reach statistical significance (*p* = 0.33) [38]. A more comprehensive interpretation is not possible as baseline ICIQ-FLUTSsex scores were not published [38] or available through an attempt at contact with the author. As such an accurate assessment of the starting degree of impact of OAB on sexual health within this study cohort is not possible, which is imperative in interpreting this data.

## Discussion

This study is important in bringing to the fore a neglected aspect of OAB research – the impact of pharmacotherapy on sexual function in female patients with OAB. Due to heterogeneity in the proportion of female participants included in the RCTs, and the concerns with reporting secondary outcome assessments, no meta-analysis is currently feasible. Studies using the FSFI were NSTIs, which lack a standard approach to synthesizing data. Although an improvement in sexual function was seen, the significance of this remains to be fully confirmed.

There is a growing recognition for equal representation in medical literature as recent societal influence has rightfully moved us into a greater consciousness over equity within a broad context. One area of recognisable deficiency has been concerning assessing sexual outcomes of conditions and their treatments in all sexes and/or genders. As sexual health conditions are often not readily disclosed (due to upbringing, embarrassment, or lack of opportunity) it is essential to design trials where these impacts are recognised.

This clinical enquiry used the robust undertaking of a systematic review methodology to investigate the changes in female sexual function scores in female patients undergoing pharmacotherapy in the management of OAB. This was done to provide an evidence-based assessment of the current data, using a transparent and reproducible search strategy with a quality of assessment done to ensure that a critical appraisal of the evidence was performed. Three databases were used in conducting this systematic review, with over 2000 abstracts screened by two reviewers ensuring a comprehensive and independently verified result was achieved.

This systematic review aimed to provide insight into the sexual health improvements in female patients with OAB, which is known to have an adverse effect on sexual health, undergoing pharmaceutical treatment. As *Kubota Y, *et al*.* succinctly stated, “since the primary goal of OAB treatment is to reduce symptoms, the final goal is to improve HRQOL,” of which sexual function is undoubtedly important [55]. Patient goal setting is valuable in clinical practice, helping doctors meet the expectations and needs of their patients, which was highlighted by *Cartwright, R *et al. [51]. Although most of the studies reviewed showed an improvement in sexual function or HRQOL within this domain (personal relationships), the evidence is weak, with significant bias introduced as these health-related quality of life metrics remain, for the most part, a secondary outcome which were inconsistently and often poorly reported on [30, 32, 37, 40, 42, 62].

A secondary aim was to establish whether there were certain medication-specific improvements which would ensure a more tailored approach to prescribing medications for patients with OAB in line with the patient’s desired or required sexual function improvements. The FSFI breaks the female sexual function experience into different domains, which would allow for discernment of domain-specific improvements with certain classes of medications and has been widely utilised [59, 60, 69, 70, 78]. Although some have questioned the usefulness of conceptualising female sexual function into different domains, the FSFI has been externally validated, is sensitive to change and does provide a female-specific assessment tool [67, 79]. It is limited in patients who have reduced sexual activity [68]. The limitation of the studies using the FSFI was due to the study designs which had inherent baseline confounding and as such limited value in evidence weighting [59, 60, 69, 70, 78]. The secondary aim was as such unable to be determined.

The King’s Health Questionnaire provides an insight into sexual health through the domain of personal relationships [44–46]. This tool’s drawbacks include a lack of discretionary capacity with regards to which aspects of female sexual function are impacted, it does not account for biological differences in sexual experience, it is not appropriate for those who are not in a family, and those who are not in a current relationship (which may be due to OAB or by choice). A further point which does need clarification is that most authors define a minimum important clinical difference (MID most defined as ≥ 5) which is the threshold where benefit for the patient is experienced [31, 34, 51]. This is important as even though the threshold for statistical significance may not be met, a patient may meet the MID and as such appreciate an improvement [34, 44].

The articles using the KHQ, largely funded by industry, have done little to provide certainty on the impact and outcomes of female patients who have sexual dysfunction because of their OAB, despite this questionnaire having been widely used in large, multicentre, double-blinded, RCTs. It is noteworthy the differences in explaining the decreased completion of the personal relationships’ domain in the KHQ forms which include embarrassment, not sexually active, or not in a relationship, with a particularly notable option for answering the sexual domain questions as “not applicable” [53, 56, 62]. In responding to health-related questionnaires *Abrams P, *et al., noted that there are limitations as patients may not respond to personal or sensitive topics such as sexual or personal relationships [31]. A further important consideration is that even if renewed sexual interest or desire does occur, confidence to proceed with entering into a sexual relationship would precede entering into a sexual relationship (and hence reported sexual satisfaction) which may introduce a time-dependent bias due to standard 12 week assessment time frame used in most studies to assess primary and secondary outcome changes [31].

An important observation by *Sand P, *et al*.,* was that KHQ domains with the greatest improvement at completion of treatment were those with the highest KHQ domain scores (i.e., greatest impairment) at baseline [62]. This is highlighted by *Schiavi MC, *et al*.,* although limited as a retrospective analysis, in that their cohort had a substantial impairment in personal relationships at baseline [58]. This encourages baseline sexual function reporting, as improvement experienced by a patient with a good baseline sexual function would presumably be minimal with treatment.

An observation by *Hsiao SM, *et al*.* showed that personal relationships, emotional domain, physical limitations, and social limitations were among the biggest predictors of therapy completion with solifenacin [49]. This may suggest the importance of addressing these needs and expectations when initiating pharmacotherapy. Similarly, *Cordozo L, *et al*.,* showed that if HRQOL or patient-reported outcomes were met, patients were less likely to request dose escalation [56].

High discontinuation rates have been noted over the long term with up to a quarter within active study participation discontinuing treatment, with very likely higher rates of discontinuation in real-world settings being observed [80]. Most respondents in a study by *Benner JS, *et al*.* reported discontinuation due to unmet expectations with regard to treatment efficacy and/ or tolerability [80]. Without well-designed trials, we remain unaware if not meeting the need for desired sexual health improvements is a contributing reason which leads to patient dissatisfaction and discontinuation with pharmacotherapy.

At the time of undertaking this systematic review, the search strategy and results acquired were shown to be the most comprehensive review on the topic. *Levy G, *et al*.* published a systematic review on sexual function outcomes concerning pelvic floor muscle training, pharmacotherapy, intravesical Botulin toxin injections and neuromodulation [81]. Although this is a good summary, the major limitations of this review include that it is not comprehensive (only one database was utilised), there was no assessment of bias or comment on the quality of studies included being made available [81]. This review, in contrast, focussed on pharmacotherapy and as such could follow more closely the requirements for a systematic review as proposed and guided by the AMSTAR 2 appraisal tool [23]. Findings were largely congruent between the two reviews.

A limitation of this review included requiring data on secondary outcomes that were often poorly recorded and/or reported on in the studies used. This review as such had to extend the inclusion of selection criteria of studies to include NSTIs which are subject to inherent baseline confounding. Although using the personal relationships’ domain of KHQ to assess sexual function outcomes may well be scrutinized, the benefit is clear in that it has been shown that, for the most part, published literature has failed to incorporate an appropriate assessment tool for outcomes in a condition which impacts female patients disproportionately and is known to impact sexual function in a large subset of patients. The questionnaires available to assess sexual function do not take sexual minority women into account. The questions should therefore be adjusted to apply to the patient’s sexual orientation to ensure a reliable assessment of sexual function was done.

The strict timetable of the academic program for which this review was conducted, limited the opportunity for more intensive engagement with authors to obtain the unreported data in the studies reviewed. Had additional data been secured, this may have enabled better evidence for the review.

Strengths of this study include that it used the AMSTAR 2 appraisal tool as a guiding framework, assisting in ensuring that this met the criteria for a good quality systematic review. In addition, multiple databases were searched, two reviewers screened abstracts, selected full-text studies for inclusion, and did data extraction and risk of bias assessment, which ensured independent scrutiny was applied thereby reducing the risk of bias in each subsequent phase of the review.

At the time of final full-text inclusion, this review included all relevant studies where the authors felt that this question could be answered, or at least answered in part. The use of multiple databases and the breadth of the articles incorporated for abstract screening ensure that there is sufficient reason to presume that all appropriate literature has been incorporated and assessed.

## Conclusion

Treatment with anticholinergic or beta-3-adrenergic receptor agonist medication for OAB may improve sexual function in females, likely benefitting those with a higher degree of bother at baseline. GRADE certainty rating: very low certainty [27, 82].

Research plays a pivotal role in informing clinical practice in both clinical enquiry and treatment. From this review, it is evident that the sexual and relationship impact of OAB in female patients is more likely to be neglected on enquiry. Although this may be due to embarrassment on behalf of participants, a more pragmatic explanation might be that trials have not been designed in a manner that encourages female participants to give honest reporting on their sexual health concerns and improvements concerning treatment.

This research should form the basis for a well-conducted randomized controlled study to accurately assess sexual function improvements in female patients being treated for OAB.

Physicians treating female patients with OAB should enquire about quality-of-life impact with specific enquiry into concerns within the domain of sexual health. If these are noted, a validated and appropriate scoring tool to assist in assessment (at baseline) and monitoring of outcomes (at follow-up) should be used when pharmacotherapy is being considered [27].

### Supplementary Information

**Supplementary Material 1.**

**Supplementary Material 2.**

**Supplementary Material 3.**

## Data Availability

All available figures and tables are available on reasonable request from the corresponding author. As this research made use of a predefined search strategy, as set out in the methodology section, data will be available, using a similar search strategy of the databases which were utilized for this review and should be reproducible.

## Abbreviations

ASEX: The arizona sexual experience scale
EpiLUTS: Epidemiology of LUTS study
FSFI: Female sexual function index
GRADE: Grading of recommendations assessment, development and evaluation
HRQOL: Health-related quality of life
ICIQ-FLUTSsex: International consultation on incontinence modular questionnaire – female sexual matters associated with lower urinary tract symptoms
KHQ: Kings health questionnaire
LUTS: Lower urinary tract symptoms
NSTI: Nonrandomized study of therapeutic outcomes
OAB: Overactive bladder
PICO: Patient, intervention, comparison, outcome
PRISMA: The preferred reporting items for systematic reviews and meta-analyses
RCT: Randomised control trials
RoB: Risk of bias
SQOL-F: Sexual quality of life–female questionnaire
