# Supplementary material for: The impact of pharmacotherapy on sexual function in female patients being treated for idiopathic overactive bladder: a systematic review
Source: BMC Womens Health. 2024 May 16;24:290. doi: 10.1186/s12905-024-03103-1 (PMC11097459; doi:10.1186/s12905-024-03103-1)
Supplement: Supplementary file 1 — Supplementary Material 1. [file 12905_2024_3103_MOESM1_ESM.pdf]

Risk of Bias Complete Evaluations

RoB2 Complete Evaluations

Study	1) Risk of Bias Arising from the Randomization Process				2) Risk of Bias Due to Deviations from Intended Interventions							3) Risk of Bias Due to Missing Outcome Data					4) Risk of Bias in Measurement of Data						Risk of Bias in Selection of Reported Results				Risk of Bias in Reporting Sexual Function
	a) Allocation sequence random	b) Assignment concealed until enrolled into intervention	c) Differences between groups raise concerns of bias	**Risk of Bias Judgement**	a) Participants Aware of Intervention	b) Were carers or people delivering the interventions aware	c) Were important non-protocol interventions balanced across groups	d) Were there failures in implementing intervention which could affect outcome	e) Was there non adherence to the assigned intervention	f) Was there an appropriate analysis done to estimate the effect of adhering to an intervention	**Risk of Bias Judgement**	a) Were data available for all, or nearly all of those randomized	b) Was there evidence that the outcome was not biased by missing outcome data	c) Could missingness in the data represent its true value	d) Is it likely that missingness in the data could represent its true value	**Risk of Bias Judgement**	a) Was the method of measuring the outcome	b) Could measurement of outcome differed between intervention groups	c) Were outcome assessors aware of the intervention	d) Could measurement of outcome be influenced by knowledge of intervention received	e) Is it likely that the assessment of the outcome was influenced by the knowledge of the intervention received	**Risk of Bias Judgement**	a) Were the data that produced this result done in accordance with a pre-specified analysis plan	b) multiple eligible outcome measurements within the outcome domain	c) multiple eligible analysis of outcomes	**Risk of Bias Judgement**	Joint reviewers ROBs of reported sexual function data
Jünemann [2006]	PY	PY	PN	Low	PN	PN	NA	PN	NA	NA	Low	PN	PN	PY	PN	Some concern	N	NI	**PN**	NA	PN	Some concern	PY	NI	NI	Some concern	High
Kelleher [2005]	PY	PY	PN	Low	PN	PN	NA	NA	NA	NA	Low	PN	PN	PY	PN	Some concern	N	N	PN	NA	NA	Low	PN	PY	PY	Some concern	High
Abrams [2008]	PY	PY	PN	Low	PN	PN	NA	NA	NA	NA	Low	**N**	PN	PY	PN	Some concern	N	**PN**	N	NA	NA	Low	N	**PN**	N	Some concern	High
Choo [2008]	Y	Y	N	Low	N	N	NA	NA	NA	NA	Low	PY	NA	NA	NA	Low	N	N	N	NA	NA	Low	Y	N	N	Low	High
Homma [2008]	PY	PY	PN	Low	PN	PN	NA	NA	NA	NA	Low	N	PN	PY	PY	High	N	N	PN	NA	NA	Low	Y	Y	Y	High	High

Study	**1) Risk of Bias Arising from the Randomization Process**				**2) Risk of Bias Due to Deviations from Intended Interventions**							**3) Risk of Bias Due to Missing Outcome Data**					**4) Risk of Bias in Measurement of Data**						**Risk of Bias in Selection of Reported Results**				**Risk of Bias in Reporting Sexual Function**
	a) Allocation sequence random	b) Assignment concealed until enrolled into intervention	c) Differences between groups raise concerns of bias	**Risk of Bias Judgement**	a) Participants Aware of Intervention	b) Were carers or people delivering the interventions aware	c) Were important non-protocol interventions balanced across groups	d) Were there failures in implementing intervention which could affect outcome	e) Was there non adherence to the assigned intervention	f) Was there an appropriate analysis done to estimate the effect of adhering to an intervention	**Risk of Bias Judgement**	a) Were data available for all, or nearly all of those randomized	b) Was there evidence that the outcome was not biased by missing outcome data	c) Could missingness in the data represent its true value	d) Is it likely that missingness in the data could represent its true value	**Risk of Bias Judgement**	a) Was the method of measuring the outcome	b) Could measurement of outcome differed between intervention groups	c) Were outcome assessors aware of the intervention	d) Could measurement of outcome be influenced by knowledge of intervention received	e) Is it likely that the assessment of the outcome was influenced by the knowledge of the intervention received	**Risk of Bias Judgement**	a) Were the data that produced this result done in accordance with a pre-specified analysis plan	b) multiple eligible outcome measurements within the outcome domain	c) multiple eligible analysis of outcomes	**Risk of Bias Judgement**	Joint reviewers ROBs of reported sexual function data
Kelleher [2008]	**PY**	**PY**	N	Low	**PN**	**PN**	NA	NA	NA	NA	Low	PY	NA	NA	NA	Low	N	**PN**	**PN**	NA	NA	Low	N	N	N	Some concern	Some concern
Rogers [2008]	Y	Y	N	Low	N	**PN**	NA	NA	NA	NA	Low	PN	PN	PY	PN	Some concern	N	**N**	**N**	NA	NA	Low	Y	N	N	Low	Low
Homma [2009]	Y	Y	N	Low	N	N	NA	NA	NA	NA	Low	PY	NA	NA	NA	Low	N	N	N	NA	NA	Low	Y	Y	N	High	High
Sand [2009]	**PY**	**PY**	**PN**	Low	**PN**	**PN**	NA	NA	NA	NA	Low	N	**PN**	**PY**	Y	High	N	**N**	N	NA	NA	Low	**N**	N	**NI**	**Some concern**	High
Van Kerrebroeck [2009]	**PY**	PY	PN	Low	N	N	NA	NA	NA	NA	Low	**PN**	N	Y	PY	High	N	N	N	NA	NA	Low	Y	PN	PY	High	High
Vardy [2009]	Y	Y	N	Low	N	N	NA	NA	NA	NA	Low	NI	N	Y	PY	High	N	N	N	NA	NA	Low	Y	N	N	Low	Some concern
Cartwright [2011]	Y	Y	PN	Low	N	N	NA	**PN**	NA	NA	Low	N	PN	PY	PN	Some concern	N	N	N	NA	NA	Low	**Y**	N	N	Low	High
Gotoh [2011]	Y	Y	N	Low	N	N	NA	NA	NA	NA	Low	PY	NA	NA	NA	Low	N	N	N	NA	NA	Low	Y	Y	N	High	High
Yamaguchi [2011]	PY	PY	**PN**	Low	N	N	NA	NA	NA	NA	Low	PN	PN	PY	PN	Some concern	N	N	N	NA	NA	Low	Y	N	PN	Low	Some concern
Park [2014]	Y	Y	PN	Low	N	N	NA	NA	NA	NA	Low	PN	PY	NA	NA	Low	N	N	N	NA	NA	Low	**N**	N	**Y**	High	Some concern

Study	1) Risk of Bias Arising from the Randomization Process				2) Risk of Bias Due to Deviations from Intended Interventions							3) Risk of Bias Due to Missing Outcome Data					4) Risk of Bias in Measurement of Data						Risk of Bias in Selection of Reported Results				Risk of Bias in Reporting Sexual Function
	a) Allocation sequence random	b) Assignment concealed until enrolled into intervention	c) Differences between groups raise concerns of bias	**Risk of Bias Judgement**	a) Participants Aware of Intervention	b) Were carers or people delivering the interventions aware	c) Were important non-protocol interventions balanced across groups	d) Were there failures in implementing intervention which could affect outcome	e) Was there non adherence to the assigned intervention	f) Was there an appropriate analysis done to estimate the effect of adhering to an intervention	**Risk of Bias Judgement**	a) Were data available for all, or nearly all of those randomized	b) Was there evidence that the outcome was not biased by missing outcome data	c) Could missingness in the data represent its true value	d) Is it likely that missingness in the data could represent its true value	**Risk of Bias Judgement**	a) Was the method of measuring the outcome	b) Could measurement of outcome differed between intervention groups	c) Were outcome assessors aware of the intervention	d) Could measurement of outcome be influenced by knowledge of intervention received	e) Is it likely that the assessment of the outcome was influenced by the knowledge of the intervention received	**Risk of Bias Judgement**	a) Were the data that produced this result done in accordance with a pre-specified analysis plan	b) multiple eligible outcome measurements within the outcome domain	c) multiple eligible analysis of outcomes	**Risk of Bias Judgement**	Joint reviewers ROBs of reported sexual function data
Yamaguchi [2014a]	Y	Y	N	Low	N	N	Y	NA	NA	NA	Low	N	**N**	**Y**	**N**	Some concerns	N	**N**	N	NA	NA	Low	Y	N	N	Low	High
Yamaguchi [2014b]	PY	**PY**	N	Low	N	N	NA	NA	NA	NA	Low	**N**	N	**Y**	N	Some concerns	**N**	N	N	NA	NA	Low	**N**	N	**Y**	High	High
Kuo [2015]	Y	Y	N	Low	N	N	NA	NA	NA	Y	Low	PY	NA	NA	NA	Low	N	N	**N**	NA	NA	Low	**Y**	PN	N	Low	Some concern
Song [2015]	PY	**PY**	N	Low	N	N	NA	NA	NA	NA	Low	**N**	**N**	PY	PN	Some concerns	N	N	N	NA	NA	Low	**Y**	PY	PN	**High**	High
Yamaguchi [2015]	PY	PY	PN	Low	N	N	NA	NA	NA	Y	Low	PY	NA	NA	NA	Low	N	N	N	NA	NA	Low	PY	PN	PN	Low	Some concern
Chughtai [2016]	Y	PY	PN	Low	Y	Y	PY	N	PY	PY	Some Conce rn	N	PN	Y	PY	High	N	N	Y	PY	PN	Some concern	PY	PN	PN	Low	High
Yamaguchi [2016]	**Y**	**Y**	N	Low	**N**	**Y**	PY	PN	PN	Y	Low	Y	NA	NA	NA	Low	N	N	PY	N	NA	Low	PY	PY	**N**	**High**	High
Yoshida [2018]	Y	Y	**N**	Low	N	N	NA	N	NA	Y	Low	Y	NA	NA	NA	Low	N	N	N	NA	NA	Low	PY	N	**N**	Low	Some concern
Hsiao [2019]	**Y**	**N**	**Y**	High	Y	Y	PY	N	N	PY	Low	PN	PN	PN	NA	Low	N	N	Y	PN	NA	Low	Y	N	**N**	Low	Low
Mitcheson [2019]	Y	Y	N	Low	N	N	NA	NA	NA	NA	Low	Y	NA	NA	NA	Low	N	N	N	NA	NA	Low	Y	N	N	Low	High

ROBIN-I Complete Evaluations

Study	Type	Bias Due to Confounding									Bias Due to Participant Selection						Bias Due to Classification of Intervention			
		1.1	1.2	1.3	1.4	1.5	1.6	1.7	1.8	Judgement	2.1	2.2	2.3	2.4	2.5	Judgement	3.1	3.2	3.3	Judgement
Sand [2006]	Prospective cohort	Y	N	NA	N	NA	**Y**	Y	Y	Moderate	N	NA	NA	Y	**N**	Low	Y	Y	PY	Moderate
Hajebrahimi [2008]	Prospective cohort	Y	N	**NA**	**N**	NA	**N**	**PN**	NA	Serious	**N**	NA	NA	Y	NA	Low	Y	Y	**PY**	**Moderate**
Kubota [2011]	Prospective cohort	Y	N	NA	PY	Y	**N**	**N**	**NA**	Moderate	N	NA	NA	Y	NA	Low	Y	PY	**PN**	**Low**
Cardozo [2012]	Prospective cohort	Y	N	NA	NA	NA	**N**	**PN**	**NA**	**Serious**	N	NA	NA	Y	NA	Low	Y	Y	**PN**	Low
Balachandran [2015]	Prospective cohort	Y	N	NA	N	NA	NA	N	NA	Serious	**N**	NA	NA	Y	NA	Low	Y	Y	**N**	Low
Zachariou [2017]	Prospective cohort	Y	N	NA	N	NA	N	N	NA	Serious	N	NA	NA	Y	NA	Low	Y	**Y**	**N**	Low
Schiavi [2018]	Retrospective analysis	Y	N	NA	Y	Y	**N**	PY	PY	Moderate	Y	PN	PY	Y	**N**	Serious	Y	N	PN	Moderate
Zachariou [2018]	Prospective cohort	Y	N	NA	**PY**	**Y**	N	**N**	**NA**	**Serious**	N	NA	NA	Y	NA	Low	Y	Y	PN	Low
Cakir [2019]	Case-control	Y	N	NA	PY	PY	**N**	PY	PY	Moderate	N	NA	NA	**Y**	NA	Low	N	Y	**N**	Moderate
Gubbiotti [2019]	Prospective cohort	Y	N	NA	NI	NA	NI	Y	Y	Moderate	N	NA	NA	Y	NA	Low	Y	Y	**N**	Low
Sönmez [2020]	Prospective cohort	PN	NA	NA	NA	NA	NA	NA	NA	Low	N	NA	NA	Y	NA	Low	Y	Y	PN	Low
Lin [2021]	Case-control	Y	N	NA	PN	NA	**N**	PN	NA	Serious	N	NA	NA	Y	NA	Low	PN	Y	PN	Moderate

Study	Type	Bias from Deviations from Intervention							Bias Due to Missing Data						Bias in Outcomes Measurements				
		4.1	4.2	4.3	4.4	4.5	4.6	Judgement	5.1	5.2	5.3	5.4	5.5	Judgement	6.1	6.2	6.3	6.4	Judgement
Sand [2006]	Prospective cohort			NA	Y	PN	PY	Moderate	**N**	N	**PN**	NA	**PY**	Moderate	**N**	Y	NA	PN	Moderate
Hajebrahimi [2008]	Prospective cohort			NA	Y	**Y**	NA	Low	**Y**	N	N	NA	NA	Low	**PY**	Y	NA	**N**	Moderate
Kubota [2011]	Prospective cohort			NA	**Y**	**Y**	NA	Low	**Y**	**N**	**N**	NA	NA	Low	**PY**	Y	NA	N	Moderate
Cardozo [2012]	Prospective cohort			NA	PY	PN	N	Serious	**Y**	**N**	**N**	NA	NA	Low	N	**PY**	NA	N	Moderate
Balachandran [2015]	Prospective cohort			**N**	**PY**	N	N	Serious	Y	**N**	**N**	NA	NA	Low	**N**	Y	NA	N	Moderate
Zachariou [2017]	Prospective cohort			N	Y	**PY**	N	Serious	PN	N	N	Y	PY	Moderate	PY	Y	**PY**	**N**	Moderate
Schiavi [2018]	Retrospective analysis			PN	**PY**	**PY**	PY	Moderate	Y	N	N	NA	NA	Low	**N**	Y	Y	N	Moderate
Zachariou [2018]	Prospective cohort			**N**	Y	**PY**	**N**	Serious	PN	N	N	N	N	Serious	**PY**	Y	Y	**N**	**Moderate**
Cakir [2019]	Case-control			PN	PY	PY	Y	Moderate	Y	N	N	NA	NA	Low	PY	Y	Y	N	Moderate
Gubbiotti [2019]	Prospective cohort			NA	Y	Y	NA	Low	Y	N	N	NA	NA	Low	**N**	Y	**NA**	N	Moderate
Sönmez [2020]	Prospective cohort			PY	Y	**PY**	NA	Low	Y	N	N	NA	NA	Low	PY	Y	Y	**N**	Moderate
Lin [2021]	Case-control			N	N	**PY**	Y	Moderate	Y	**N**	**N**	NA	NA	Low	PY	Y	Y	**N**	Moderate

Study	Type	Bias in Selection of Reported Results				Risk of Bias in Reporting Sexual Function
		**7.1**	**7.2**	**7.3**	**Judgement**	**1.**
Sand [2006]	Prospective cohort	N	**PN**	**PY**	Low	Moderate
Hajebrahimi [2008]	Prospective cohort	N	N	N	Low	Low
Kubota [2011]	Prospective cohort	N	**PY**	N	Moderate	Serious
Cardozo [2012]	Prospective cohort	N	N	N	Low	Moderate
Balachandran [2015]	Prospective cohort	N	N	N	Low	Moderate
Zachariou [2017]	Prospective cohort	N	N	N	Low	Low
Schiavi [2018]	Retrospective analysis	N	N	N	Low	Moderate
Zachariou [2018]	Prospective cohort	N	Y	N	Moderate	Low
Cakir [2019]	Case-control	N	N	N	Low	Low
Gubbiotti [2019]	Prospective cohort	N	N	N	Low	Low
Sönmez [2020]	Prospective cohort	N	N	N	Low	Low
Lin [2021]	Case-control	N	N	**Y**	**Moderate**	Moderate

Study	Summary Table								
	**1**	**2**	**3**	**4**	**5**	**6**	**7**	**8**	
Sand [2006]	M	L	M	M	M	M	L	M	Underreporting of PR
Hajebrahimi [2008]	S	L	M	L	L	M	L	L	Data complete
Kubota [2011]	M	L	L	L	L	M	M	S	Data missing; only graphic representation
Cardozo [2012]	S	L	L	S	L	M	L	M	No baseline
Balachandran [2015]	S	L	L	S	L	M	L	M	Multiple analysis
Zachariou [2017]	S	L	L	S	M	M	L	L	Data complete
Schiavi [2018]	M	S	M	M	L	M	L	M	Multiple analysis; retrospective
Zachariou [2018]	S	L	L	S	S	M	M	L	Data complete
Cakir [2019]	M	L	M	M	L	M	L	L	Data complete
Gubbiotti [2019]	M	L	L	L	L	M	L	L	Data complete
Sönmez [2020]	L	L	L	L	L	M	L	L	Data complete
Lin [2021]	S	L	M	M	L	M	M	M	Missing data
