# Supplementary material for: The impact of pharmacotherapy on sexual function in female patients being treated for idiopathic overactive bladder: a systematic review
Source: BMC Womens Health. 2024 May 16;24:290. doi: 10.1186/s12905-024-03103-1 (PMC11097459; doi:10.1186/s12905-024-03103-1)
Supplement: Supplementary file 2 — Supplementary Material 2. [file 12905_2024_3103_MOESM2_ESM.pdf]

Communication Log with Study Authors

Author	Year	Title	Emailed	Response
Abrams	2008	Clinical relevance of health-related quality of life outcomes with darifenacin	mohamed-heor.omar@novartis.com	Address not found
Van Kerrebroek	2009	Correlations among improvements in urgency urinary incontinence, health-rel	p.vankerrebroeck@mumc.nl	Email blocked
Balachandran	2015	Third-line treatment for overactive bladder: should mirabegron be tried befor	aswini@doctors.net.uk	No response
Cardozo	2012	Safety and efficacy of flexible-dose fesoterodine in British subjects with overa	linda@lindacardozo.co.uk	Data not available
Gotoh	2011	Propiverine hydrochloride in Japanese patients with overactive bladder: A ran	gotoh@med.nagoya-u.ac.jp	No response
Homma	2009	A randomized, double-blind, placebo- and propiverine-controlled trial of the r	homma-uro@umin.ac.jp	No response
Homma	2008	A randomized, double-blind, placebo-controlled phase II dose-finding study of	homma-uro@umin.ac.jp	No response
Kubota	2011	Correlation Between Improvements in Overactive Bladder Symptom Score an	yasuekbt@med.nagoya-cu.ac.jp	Interested and wants to help, can't currently access data
Kuo	2015	Results of a Randomized, Double-Blind, Parallel-Group, Placebo- and Active-C	hck@tzuchi.com.tw	Data file deleted
Mitcheson	2019	Vibegron (RVT-901/MK-4618/KRP-114V) Administered Once Daily as Monothe	tara_frenkl@merck.com	Address not found
Choo	2008	Efficacy and safety of solifenacin succinate in Korean patients with overactive	jeongkl@kumc.or.kr	No response
Sand	2007	Oxybutynin transdermal system improves the quality of life in adults with ove	p-sand@northwestern.edu	Address not found
Yamaguchi	2011	Efficacy, Safety and Tolerability of Fesoterodine in Asian Patients with Overac	yamaosa@ee.ce.nihon-u.ac.jp	Address not found
Yamaguchi	2014	Efficacy and safety of once-daily oxybutynin patch versus placebo and propiv	yamaosa@ee.ce.nihon-u.ac.jp	Address not found
Yamaguchi	2014	Phase III, randomised, double-blind, placebo-controlled study of the β3-adren	yamaosa@ee.ce.nihon-u.ac.jp	Address not found
Yamaguchi	2015	Efficacy and Safety of the Selective β3-Adrenoceptor Agonist Mirabegron in J	yamaosa@ee.ce.nihon-u.ac.jp	Address not found
Yoshida	2018	Vibegron, a Novel Potent and Selective b3-Adrenoreceptor Agonist, for the Tr	akko-maki@umin.net	No response
Payne	2007	Redefining response in overactive bladder syndrome	cpayne@stanford.edu	No response
Park	2014	A randomised, prospective double-blind, propiverine-controlled trial of imidaf	mschoo@amc.seoul.kr	Data with drug company
Cartwright	2010	Patient-selected goals in overactive bladder: a placebo controlled randomized	rufus.cartwright@gmail.com	No response
Jünemann	2006	Propiverine Hydrochloride Immediate and Extended Release: Comparison of E	kjuenemann@urology.uni-kiel.de	No response
Kelleher	2008	Impact of fesoterodine on quality of life: pooled data from two randomized t	Con.Kelleher@gstt.nhs.uk	Data with Pfizer
Rogers	2008	Efficacy of tolterodine on overactive bladder symptoms and sexual and emot	RRogers@salud.unm.edu	Email blocked
Sand	2009	Trospium chloride extended release is effective and well tolerated in women	psand@northshore.org	No response
Song	2015	The efficacy and tolerability of tarafenacin, a new muscarinic acetylcholine re	mshoo@amc.seoul.kr	No response
