# Supplementary material for: The impact of pharmacotherapy on sexual function in female patients being treated for idiopathic overactive bladder: a systematic review
Source: BMC Womens Health. 2024 May 16;24:290. doi: 10.1186/s12905-024-03103-1 (PMC11097459; doi:10.1186/s12905-024-03103-1)
Supplement: Supplementary file 3 — Supplementary Material 3. [file 12905_2024_3103_MOESM3_ESM.pdf]

FSFI Domain Specific Results

1. Desire

Study	Design	Control	Drug	Dose	N treated	Baseline		Post treatment		
						Desire	SD	Desire	SD	P-value
Zachariou [2017]	Prospective cohort	OAB	Tolterodine	ER 4mg	85	2.5	0.2	4.5	0.2	<0.01
Cakir [2019]	Case-control	Healthy	Anticholinergics		216	2.94	0.69	3.19	0.61	<0.01
Lin [2021]	Case-control	Healthy	Tolterodine	2mg b.d.	55	2.07	0.88	3.05	0.96	<0.001
Zachariou [2018]	Prospective cohort	OAB	Mirabegron	50mg	35	3	1.2	4.8	1.2	<0.001
Gubbiotti [2019]	Prospective cohort	None	Mirabegron	50mg	50	3.03	0.65	3.51	0.88	0.04

2. Arousal

Study	Design	Control	Drug	Dose	N treated	Baseline		Post treatment		
						Arousal	SD	Arousal	SD	P-value
Zachariou [2017]	Prospective cohort	OAB	Tolterodine	ER 4mg	85	3.1	0.2	4.4	0.3	<0.01
Cakir [2019]	Case-control	Healthy	Anticholinergics		216	3.43	0.89	3.85	0.74	<0.01
Lin [2021]	Case-control	Healthy	Tolterodine	2mg b.d.	55	1.96	1.39	3.14	0.88	<0.001
Zachariou [2018]	Prospective cohort	OAB	Mirabegron	50mg	35	3	0.8	4.8	0.9	<0.001
Gubbiotti [2019]	Prospective cohort	None	Mirabegron	50mg	50	2.87	0.61	3.35	0.94	<0.0001

3. Lubrication

Study	Design	Control	Drug	Dose	N treated	Baseline		Post treatment		
						Lubrication	SD	Lubrication	SD	P-value
Zachariou [2017]	Prospective cohort	OAB	Tolterodine	ER 4mg	85	3.4	0.3	4.3	0.3	<0.01
Cakir [2019]	Case-control	Healthy	Anticholinergics		216	4.19	0.62	4.09	0.56	0.063
Lin [2021]	Case-control	Healthy	Tolterodine	2mg b.d.	55	2.56	1.94	3.59	1.3	<0.001
Zachariou [2018]	Prospective cohort	OAB	Mirabegron	50mg	35	3.9	1.1	4.8	1.2	<0.001
Gubbiotti [2019]	Prospective cohort	None	Mirabegron	50mg	50	3.31	0.72	3.93	1.06	0.0008

4. Pain

Study	Design	Control	Drug	Dose	N treated	Baseline		Post treatment		
						Pain	SD	Pain	SD	P-value
Zachariou [2017]	Prospective cohort	OAB	Tolterodine	ER 4mg	85	2.4	0.2	4.6	0.4	<0.01
Cakir [2019]	Case-control	Healthy	Anticholinergics		216	4.14	0.88	4.53	0.78	<0.01
Lin [2021]	Case-control	Healthy	Tolterodine	2mg b.d.	55	2.34	1.81	3.41	1.35	<0.001
Zachariou [2018]	Prospective cohort	OAB	Mirabegron	50mg	35	3.2	0.8	4.4	1.2	<0.001
Gubbiotti [2019]	Prospective cohort	None	Mirabegron	50mg	50	3.24	0.88	3.52	0.93	0.58

5. Orgasm

Study	Design	Control	Drug	Dose	N treated	Baseline		Post treatment		
						Orgasm	SD	Orgasm	SD	P-value
Zachariou [2017]	Prospective cohort	OAB	Tolterodine	ER 4mg	85	3.5	0.3	4.5	0.3	<0.05
Cakir [2019]	Case-control	Healthy	Anticholinergics		216	3.51	0.71	4.1	0.42	<0.01
Lin [2021]	Case-control	Healthy	Tolterodine	2mg b.d.	55	2.19	0.91	2.8	0.65	<0.001
Zachariou [2018]	Prospective cohort	OAB	Mirabegron	50mg	35	3.6	0.8	4.8	1	<0.001
Gubbiotti [2019]	Prospective cohort	None	Mirabegron	50mg	50	3.36	0.74	3.8	0.96	0.0001

6. Satisfaction

Study	Design	Control	Drug	Dose	N treated	Baseline		Post treatment		
						Satisfaction	SD	Satisfaction	SD	P-value
Zachariou [2017]	Prospective cohort	OAB	Tolterodine	ER 4mg	85	2.6	0.2	4.2	0.3	<0.01
Cakir [2019]	Case-control	Healthy	Anticholinergics		216	3.26	1.01	3.68	0.89	<0.01
Lin [2021]	Case-control	Healthy	Tolterodine	2mg b.d.	55	2.1	1.34	2.82	1	<0.001
Zachariou [2018]	Prospective cohort	OAB	Mirabegron	50mg	35	3.2	0.8	4	0.8	<0.001
Gubbiotti [2019]	Prospective cohort	None	Mirabegron	50mg	50	3.33	0.91	4.04	1.05	0.0008
